# Retinal Microvascular Changes in COVID-19 Bilateral Pneumonia Based on Optical Coherence Tomography Angiography

**DOI:** 10.3390/jcm11133621

**Published:** 2022-06-23

**Authors:** Magdalena Kal, Mateusz Winiarczyk, Elżbieta Cieśla, Bernadetta Płatkowska-Adamska, Anna Walczyk, Michał Biskup, Paweł Pabjan, Stanisław Głuszek, Dominik Odrobina, Jerzy Mackiewicz, Dorota Zarębska-Michaluk

**Affiliations:** 1 Collegium Medicum, Jan Kochanowski University, 25369 Kielce, Poland; kalmagda@gmail.com (M.K.); bernadetta.platkowska@gmail.com (B.P.-A.); sgluszek@wp.pl (S.G.); dorota1010@tlen.pl (D.Z.-M.); 2 Ophthalmic Clinic, Voivodeship Hospital, 25736 Kielce, Poland; a.walczyk79@wp.pl (A.W.); michal.biskup@onet.pl (M.B.); dominik.odrobina@ujk.edu.pl (D.O.); 3 Department of Vitreoretinal Surgery, Medical University of Lublin, 20819 Lublin, Poland; jerzymackiewicz@umlub.pl; 4 Institute of Medical Science, Jan Kochanowski University, 25369 Kielce, Poland; eciesla@ujk.edu.pl (E.C.); pabjan3@tlen.pl (P.P.); 5 Department of Infectious Disease, Provincial Hospital, 25317 Kielce, Poland

**Keywords:** COVID-19, SARS-CoV-2, microvasculature changes, OCTA, optical coherence tomography, angiography, retinal microvasculature, retina, FAZ, foveal avascular zone

## Abstract

The purpose of this study was to evaluate retinal and choroidal microvascular alterations with optical coherence tomography angiography (OCTA) in COVID-19 patients hospitalized because of bilateral pneumonia caused by SARS-CoV-2. The vessel density (VD) and foveal avascular zone (FAZ) of 63 patients with SARS-CoV-2 pneumonia who had positive polymerase chain reaction (PCR) tests and who recovered after receiving treatment and 45 healthy age- and gender-matched controls were evaluated and compared using OCTA in the superficial capillary plexus (SCP) and deep capillary plexus (DCP). The VD was also estimated in both groups in the choriocapillaris (CC). In COVID-19 patients, there was a statistically significant difference between the patients and a control group in both superficial (FAZs) and deep (FAZd) avascular zone (*p* = 0.000). The VD was significantly lower in the foveal area in choriocapillaris (*p* = 0.046). There were no statistically significant changes in the VD in the superior, inferior, nasal, and temporal quadrants in superficial and deep plexus, or in the choriocapillaris. The VD was not significantly lower in the foveal area in superficial or deep plexus. COVID-19 may affect the retinal vasculature, causing ischemia, enlargement of the FAZ, and lowering of the VD in the choriocapillaris area. Routine ophthalmic examination after SARS-CoV-2 infection should be considered in the course of post-infectious rehabilitation.

## 1. Introduction

Coronavirus disease 2019 (COVID-19) is caused by severe acute respiratory syndrome coronavirus 2 (SARS-CoV-2), a new beta coronavirus that was first identified in December 2019 in Wuhan, China. Since then, SARS-CoV-2 has spread as a pandemic, announced by the World Health Organization (WHO) on 11 March 2020. Transmission of the virus occurs primarily by droplets, through exposure to respiratory secretions containing the pathogen, but infection through contact with contaminated objects or surfaces was also reported [1].

The clinical picture of the disease ranges from asymptomatic through mild and moderate respiratory infection to life-threatening viral pneumonia with acute respiratory distress syndrome, septic shock, and multiple organ dysfunction with a very high mortality rate. SARS-CoV-2 uses a spike protein that directly binds with a human angiotensin-converting enzyme 2 (ACE2) in order to infect human cells [2,3,4]. ACE2 is ubiquitously expressed in the central nervous system (CNS), blood vessels, lungs, kidneys, enterocytes, nose, liver, and the immune system [5,6]. In the human eye, ACE2 was detected in the cornea, conjunctiva, aqueous humor, and retina [7,8].

The virus has been detected in the tears of COVID-19 symptomatic and asymptomatic patients, as well as in the retina of human cadavers, using a real-time polymerase chain reaction (PCR) [9]. Microvascular endothelial injury and cytokine overproduction (cytokine storm) were hypothesized as responsible for multiple organ failure in severe course of the disease [10]. Postmortem examinations revealed the presence of viral elements within kidney endothelial cells and the increased number of inflammatory cells in samples from other organs, suggesting endotheliitis [11].

The vascular endothelium plays a role in the control of vascular tone and the preservation of the blood–retinal barrier. Dysfunction of the endothelium can lead to microvascular ischemia due to mechanisms including vasoconstriction and inflammation [12,13]. It is possible to visualize the macular microvasculature using noninvasive OCTA. Many studies have proved that OCTA can successfully visualize the retinal microvascular network in many ocular and general diseases [14,15].

Our study aimed to assess the macular anatomy, macular vessel density, and the foveal avascular zone in patients who recovered after hospitalization due to COVID-19.

## 2. Materials and Methods

### 2.1. Subjects

Cross-sectional, consecutive, and prospective case-control series. COVID-19 patients, confirmed with at least one PCR test, were selected from a group of cases with bilateral pneumonia. Patients were admitted at the Department of Infectious Diseases of WSZ in Kielce during March, April, May, and June 2021. This project was approved by the Bioethics Committee of Collegium Medicum of Jan Kochanowski University in Kielce (study codes 54 approved 1 July 2021). Written informed consent was obtained from all patients. All patients were examined 8 weeks after hospital discharge (group 1). This group consisted of 63 subjects who agreed to participate (n = 63; eyes 120) with a mean age of 51.33 ± 1.45. At the moment of ocular examination, all patients were already asymptomatic. Nineteen patients (3.16%) suffered from hypertension and three patients (4.76%) suffered from dyslipidemia. Twenty-two (34.92%) patients received oxygen therapy during hospitalization.

The control group (group 2) included healthy patients who attended the ophthalmology department for a routine eye examination. This group consisted of 45 subjects (n = 45; eyes = 83) with a mean age of 47.76 ± 1.38. Written informed consent was obtained from all patients. Inclusion criteria for this group were as follows: age of 30–70 years; negative laboratory tests for SARS-CoV-2 infection (PCR from nasopharyngeal swab); absence of COVID-19 symptoms in the past or close contact with COVID-19 patients within the 14 days before the examination; absence of concomitant eye diseases. Demographic characteristics of both groups are presented in Table 1. 

**Figure 1:**
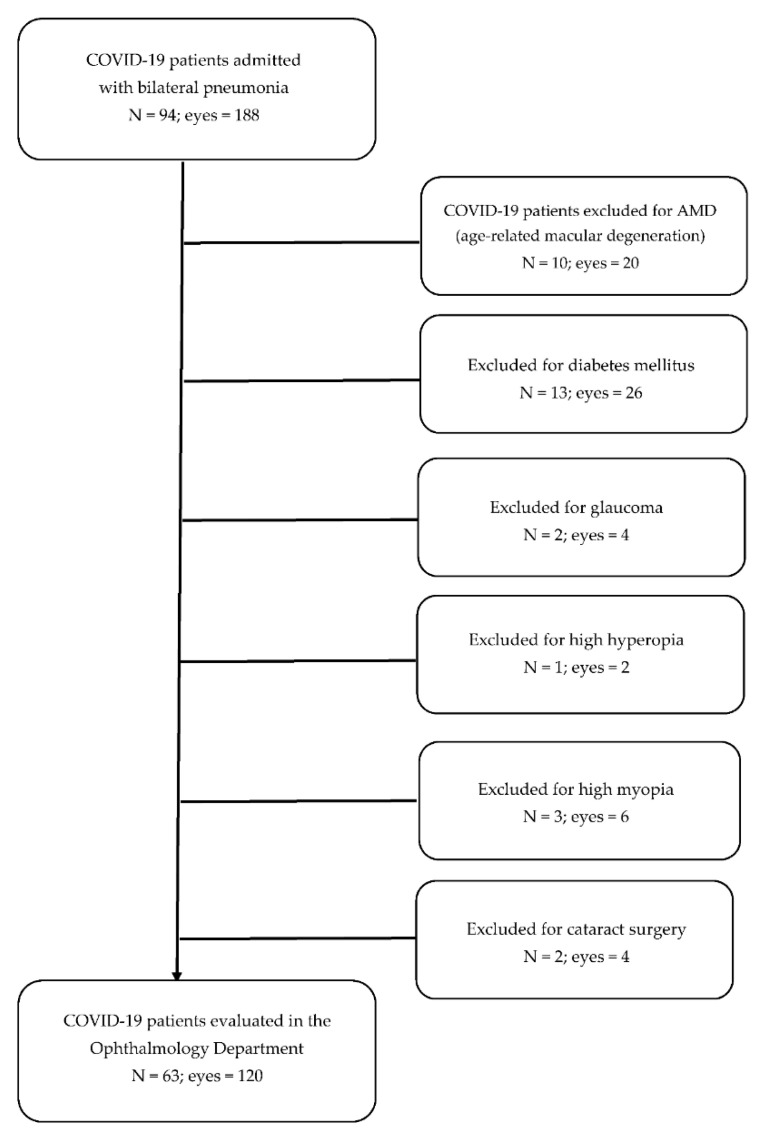
Flowchart of COVID-19 patients included in the study and excluded from the study.

A flow chart illustrating the consolidated standard for reporting all trials which describe included and excluded eyes is presented in Figure 1.

Exclusion criteria related to eye diseases for both groups were myopia >3 diopters, hyperopia >3 diopters, retinal vascular disease, macular and optic nerve disease, previous ocular surgery (including cataract or glaucoma surgery), uveitis, ocular trauma, age-related macular degeneration, other retinal degenerations and media opacity affecting the OCTA’s scan or image quality, and diabetes mellitus.

### 2.2. Ophthalmic Examination

Both groups underwent complete ophthalmic examination, including a best corrected visual acuity (BCVA) test measured on a logMAR scale, intraocular pressure (IOP) measurement, slit lamp examination, OCT of the macula and optic nerve, and angio-OCT (OCTA).

### 2.3. Optical Coherence Angiography Measurements

All scans were acquired with swept-source angio-OCT (DRI-OCT Triton SS-OCT Angio, Topcon Inc., Tokyo, Japan). OCT protocols included 3D macula 7 × 7 mm scanning protocols and 3D 6 × 6 mm disc scanning protocols. OCTA images were captured using the 4.5 × 4.5 mm and the 6 × 6 mm scanning protocols. All scans eligible for the study reached an image quality of at least 65%.

Structural OCT macular parameters were measured using the early-treatment diabetic retinopathy (ETDRS) grid centered in the fovea by manual fixation. Three areas of interest were defined as the fovea, the inner ring (IR), and the outer ring (OR). The retinal stratus and parameters analyzed were the total retina (TR), the retinal nerve fiber layer (RNFL), the ganglion cell layer (GCL), and choroid thickness, delineated automatically by the built-in segmentation software. Optic nerve head RNFL thickness was measured in each of four quadrants using a radial scan centered on the optic nerve head and presented as mean RNFL.

OCTA parameters evaluated were vessel density (VD) in the three different plexi: superficial capillary plexus (SCP), deep capillary plexus (DCP) and choriocapillaris (CC), using the ETDRS grid subfields to define the areas of interest. The mean vessel density (VD) was calculated as the average value obtained in the parafoveal area, defined as the area conformed by the inner superior (IS), inner nasal (IN), inner inferior (II), and inner temporal (IT) ETDRS subfields centered on the macula by fixation. The foveal avascular zone (FAZ) area was manually delineated on the SCP and the DCP by two independent graders, encompassing the central fovea where no clear vessels were seen in the image.

### 2.4. Statistical Analysis

All statistical analyses were performed using Statistica 13.3 (StatSoft, Kraków, Poland). Clinical demographics and imaging data were analyzed with frequency and descriptive statistics. The description of quantitative variables was performed using the mean (M), standard error of the mean (SEM), median (Me), and quartiles (IQR). Differences between COVID-19 and control groups were tested using the Mann–Whitney U test. Clinical variables, structural OCT, and OCTA parameters were compared between cases and controls using Student’s t-test. Categorical variables were presented as percentages, and comparisons between groups were performed with the chi-square test. Pearson (r) or Spearman’s correlation coefficient, Q (rho), was used to assess associations between SCP (%), DCP (%), CC (%), RNFL retinal, GCL, BMCSI, and RNFL optic nerve. A *p*-value of less than 0.05 was regarded as statistically significant. 

## 3. Results

A total of 203 eyes were included in the study: 120 COVID-19-affected patients and 83 eyes in the control group. In all, 43 men and 20 women with COVID-19 bilateral pneumonia participated in the analysis. The control group consisted of 28 men and 17 women. The mean age of the study group was 51.33 (SEM = 1.45), while the mean age of the control group was 47.76 (SEM = 1.38). 

### 3.1. Structural OCT Outcomes

In the structural OCT analysis, a statistically significant number of thicker RNFL retinas was observed in COVID-19 cases compared to controls in the inner superior ring (29.18 ± 0.24 vs. 28.35 ± 0.22, *p* = 0.024) and inner nasal ring (24.28 ± 0.21 vs. 23.69 ± 0.31, *p* = 0.018) (Supplementary Table S1). No statistically significant difference was observed in the foveal area of RNFL, GCL, the retinal thickness, or the choroidal thickness (Supplementary Table S2). In the optic nerve head analysis, a significantly thicker RNFL in the superior sector (132.49 ± 1.33 vs. 127.63 ± 1.43, *p* = 0.021) and in the inferior sector (136.42 ± 1.33 vs. 131.68 ± 2.01, *p* = 0.010) was observed in COVID-19 patients (Supplementary Table S3).

A significantly thicker GCL was observed in COVID-19 patients in the inner superior ring (122.79 ± 0.86 vs.119.66 ± 1.09, *p* = 0.012), the inner nasal ring (118.21 ± 0.89 vs. 115.41 ± 1.14, *p* = 0.023), the inner temporal ring (110.85 ± 0.86 vs.108.72 ± 1.0, *p* = 0.023), and the outer inferior ring (105.72 ± 0.90 vs. 102.65 ± 1.06, *p* = 0.029) (Supplementary Table S4). 

Detailed information about retinal thickness measurements can be found in supplementary tables (Supplementary Tables S1–S4)

### 3.2. OCT Angiography Outcomes

**Figure 2:**
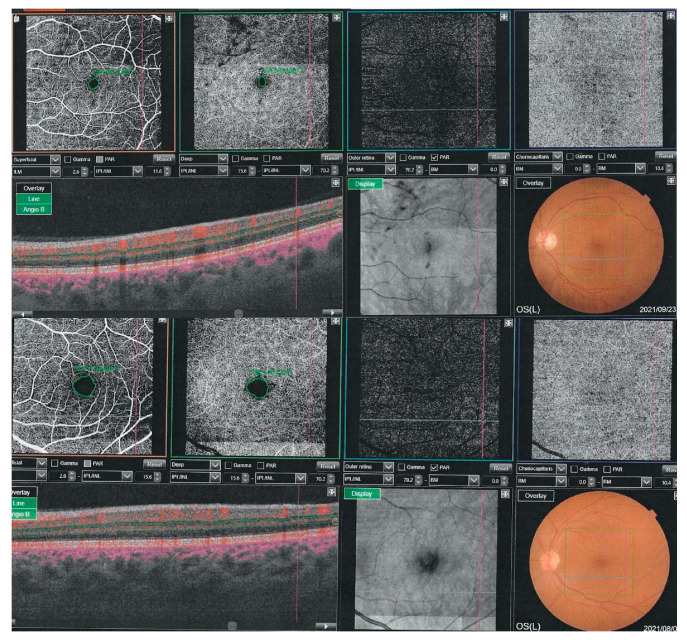
Representative image of the foveal avascular zone (FAZ) visible on the OCT in the control group (upper image) and the COVID-19 group (lower image). FAZ enlargement can be seen in the COVID-19 group.

We observed that the FAZ area was significantly larger in COVID-19 patients than in controls both for the SCP (326.81 ± 9.77 vs. 251.03 ± 1.10, *p* = 0.000) and for the DCP (357.76 ± 13.17 vs. 237.12 ± 11.88, *p* = 0.000) (Table 2). Enlargement of the FAZ area is presented in Figure 2. 

The VD was significantly lower in the foveal area of CC (5.42 ± 0.38 vs. 5.43 ± 0.47, *p* = 0.046) (Table 3), but there were no significant differences in the parafoveal area in this plexus (66.86 ± 0.12 vs. 66.91 ± 0.20, *p* = 0.456). 

No significant differences were found in VD in the parafoveal area (51.95 ± 0.22 vs. 51.84 ± 0.28, *p* = 0.620) (Supplementary Table S5) and in the foveal area (20.69 ± 0.39 vs. 20.70 ± 0.45, *p* = 0.706) (Supplementary Table S6). 

In the DCP, no significant differences were found in VD in the parafoveal area (54.29 ± 0.24 vs. 53.86 ± 0.27, *p* = 0.241) (Supplementary Table S5) and in the foveal area (17.17 ± 0.43 vs. 16.93 ± 0.49, *p* = 0.849) (Supplementary Table S6).

There were no statistically significant differences in the superior, inferior, temporal, or nasal areas in the SCP, DCP, or CC between COVID-19 patients and the control group. Detailed information can be found in Supplementary Tables S6–S9. 

### 3.3. Differences in the OCT-A Results according to Sex

We observed that the FAZ area was significantly larger in female COVID-19 patients than in male COVID-19 patients in the SCP (377.81 ± 19.65 vs. 303.02 ± 10.28, *p* < 0.00). 

The VD in the SCP was significantly lower in women than in men in COVID-19 patients in the foveal area of the SCP (18.48 ± 0.77 vs. 21.72 ± 0.40, *p* < 0.001), but significantly higher in women than in men in the inferior area of the SCP (47.70 ± 0.90 vs. 46.98 ± 0.42, *p* < 0.039). 

The VD in the DCP was significantly lower in women than in men in COVID-19 patients in the superior area (50.99 ± 0.60 vs. 52.43 ± 0.33, *p* = 0.009), in the nasal area (48.51 ± 0.57 vs. 49.26 ± 0.30, *p* = 0.043) and in the temporal area (46.87 ± 0.44 vs. 47.93 ± 0.27, *p* = 0.036).

In the structural OCT analysis, a statistically significantly thicker RNFL retina was observed in men than in women in COVID-19 cases in the foveal area (2.92 ± 0.35 vs.3.88 ± 0.29, *p* = 0.023), in the inner nasal ring (24.54 ± 0.26 vs. 23.65 ± 0.36, *p* = 0.022), in the inner inferior ring (29.94 ± 0.42 vs. 28.59 ± 0.41, *p* = 0.007), in the inner temporal ring (20.71 ± 0.26 vs. 18.54 ± 0.43, *p* < 0.001), and in the outer temporal ring (23.60 ± 0.46 vs. 22.03 ± 0.51, *p* < 0.022).

No statistically significant difference was observed in the RNFL optic nerve between men and women in COVID-19 patients or in choroidal thickness (BMCSI). 

A significantly thicker GCL was observed in men than in women in COVID-19 patients in the foveal area (52.62 ± 1.32 vs. 46.16 ± 1.59, *p* = 0.002), in the inner temporal ring (112.34 ± 1.03 vs.107.08 ±1.39, *p* = 0.002), and in the outer temporal ring (94.22 ± 0.86 vs. 90.54 ± 1.33, *p* = 0.005).

The retinal thickness was significantly thicker in men than in women in COVID-19 patients in the foveal area (249.45 ± 2.93 vs. 236 ± 3.25, *p* < 0.001), in the inner superior ring (321.23 ± 1.78 vs. 312.32 ± 2.54, *p* = 0.001), in the inner nasal ring (320.87 ± 1.87 vs. 311.08 ± 2.94, *p* = 0.005), in the inner inferior ring (318.10 ± 1.67 vs. 309.19 ± 2.72), *p* = 0.002), in the inner temporal ring (308.50 ± 2.30 vs. 295.76 ± 2.86, *p* < 0.001), in the outer inferior ring (267.65 ± 1.44 vs. 262.78 ± 2.19, *p* = 0.023), and in the outer temporal ring (262.22 ± 1.88 vs. 254.43 ± 2.08, *p* < 0.001).

Detailed information on the retinal thickness measurements can be found in Supplementary Table S10.

## 4. Discussion

There is currently growing evidence regarding the impairment of retinal microcirculation during SARS-CoV-2 infection due to vasculitis and thromboembolism [16,17]. The OCT and OCTA are valuable tools in the objective estimation of a retinal microvasculature state in COVID-19 patients and obtained parameters can be used as potential biomarkers of vascular damage in other organs. The retinal microvasculature can be also estimated in vivo [18,19,20].

The available literature describes many retinal changes due to COVID-19 infection. It is not known whether these features are secondary to the virus’ presence. Many researchers consider the microvascular and immunological processes secondary to the viral infection in the respiratory system [21,22].

Araujo-Silva et al. described the results of retina biopsy from enucleated eyes of three dead patients with confirmed COVID-19 disease with more than 50% lung area affected. The images obtained by transmission electron microscopy visualized viral particles in the inner and outer nuclear layer (INL and ONL). The immunofluorescence microscopy confirmed two essential proteins of the SARS-CoV-2 viral particles: S protein, exposed on the virus surface, and nucleocapsid protein located within the virus. The nucleocapsid protein was visualized in the ganglion layer (GCL) [23]. These findings confirm the penetration of the virus through the blood–retinal barrier and therefore can damage the retinal structure both in the course of the primary infection, and as a later complication.

Below we discuss the most important findings of our work regarding changes ongoing in the retinal microvasculature in COVID-19-affected patients. 

### 4.1. Foveal Avascular Zone (FAZ) Enlargement

One of the most prominent features in our study was the enlargement of the FAZ area in the SCP. There are conflicting data in the literature, as some authors confirm this finding [24], while no significant differences were noted by others [25,26,27]. We observed significantly greater FAZ area in the deep plexus in infected patients, contrary to other studies where no such observation was made [24,27]. 

Enlargement of the FAZ zone can be a consequence of retinal ischemia due to endothelial dysfunction, vasoconstriction, and procoagulant activity. It can be the perfusion deficit in the foveal area caused by general hypoxia and inflammation.

Postmortem studies confirmed the presence of microvascular thrombosis, endotheliitis, viral elements, apoptotic bodies, and inflammatory cells within the endothelium of small vessels in many organs. The ischemic processes can cause enlarging of this FAZ area. It can be observed in many vascular diseases such as diabetes mellitus or retinal vascular occlusion [28,29], and can be the factor for gradual vision loss in long-term follow-up. 

### 4.2. Thickening of Ganglion Cell Layer (GCL)

The ganglion cell layer (GCL) and the inner plexiform layer (IPL) are supplied with blood by the SCP, but the DCP supplies the outer plexiform layer (OPL) adjacent to the outer nuclear layer (ONL). The OPL includes oxygen-dependent synapses of photoreceptors, bipolar cells, and horizontal cells. The SCP and the DCP are the final branches of the central retinal artery. We can expect the ischemia and secondary atrophy of the inner retina due to endotheliitis and micro thrombotic processes within these small vessels.

In our group, we did not find a difference between COVID-19 patients and the control group in the foveal GCL layer, but we confirmed significantly thicker GCL in the inner superior ring, the inner nasal ring, the inner temporal ring, and the outer inferior ring. Our results are in accordance with the study performed by Burgos-Blasco et al. on children between 6 and 18 years who recovered from COVID-19. They observed increased macular GCL in the nasal outer and temporal inner sectors [30]. On the contrary, Jorge Gonzales-Zamora et al. observed a significantly thinner GCL in the foveal area in COVID-19 patients 14 days after hospital discharge compared to controls. The GCL layer in the inner and outer ring was not evaluated [24], which can explain this difference as well as longer follow-up time in our group. 

### 4.3. Thickening of Retinal Nerve Fiber Layer (RNFL) 

The RNFL and the GCL layers are the neuroretinal layers. John Dowling referred to the human retina as “the approachable part of the brain”, which can be clinically visualized with proper tools [31]. Many studies detected the presence of ACE2 receptors in the nervous system. SARS-CoV-2 RNA has also been identified in the cerebrospinal fluid. The hypoxia and the “cytokine storm” induced by the infection can lead to neural damage [32,33]. Patton N et al. defined the retina embryologically as an extension of the brain and there is a significant homology on the anatomy and regulatory mechanisms of their macro- and microvasculature [34]. 

In our study, the foveal RNFL was not statistically different between COVID-19 patients and the control group, but a significantly thicker RNFL retina was observed in COVID-19 cases in the inner superior ring and inner nasal ring. There were no signs of choroidal neovascularization, or any abnormalities in the RPE in these regions. It is difficult to state the origin of this change. Most authors also did not find differences in foveal RNFL [24,26,35].

Contrary to our analysis, Burgos-Blanco et al. confirmed decreased macular RNFL in the nasal outer and temporal inner sectors, but also an increase in global peripapillary in the temporal superior and temporal sectors [30]. Authors suspect that the increased peripapillary RNFL thickness can be explained by neuronal cell edema secondary to a neuroinflammatory injury similar to edema in the nervous system [30].

Savastano et al. described decreased radial peripapillary capillary plexus (RPCP) perfusion density in post-COVID-19 patients. The RNFL thickness was linearly correlated with the RPCP flow index and perfusion density in this study group. The impairment in the blood supply to the optic nerve may result in the peripapillary RNFL thinning [35].

Marinho et al. reported the presence of hyperreflective lesions at the GCL and inner plexiform layer (IPL) in adults 11-33 days after the onset of COVID-19 symptoms. Subtle cotton wool spots and microhemorrhages were present along the retinal branches [21]. In our study, we did not observe any alterations visible in the fundoscopy.

In our study, we observed significant thickening of the RNFL in the optic nerve head. This stays in line with Gonzalez-Zamora et al. findings, as they also noticed it in the superior and inferior sectors of the optic nerve head in COVID-19 patients [24].

### 4.4. Vessel Density (VD) Alterations

Another parameter analyzed in this study was the VD. We did not notice, either in the SCP or the DCP, statistically significant differences between groups in VD in the foveal or the parafoveal area. Our results are consistent with those reported by Dominika Szkodny et al. [26], but most of the studies available show a reduction in the VD, i.e., Gonzales-Zamora et al. observed reduced VD in the SCP and most of the DCP in the parafoveal and the foveal area in hospitalized groups [24]. Similar results were seen by Abrishami et al. in lower VD in the SCP and DCP in patients recovered from COVID-19 disease. They considered the direct viral infection of the retina and the secondary phenomenon of systemic inflammation [36]. Guamez-Villahoz et al. also presented significantly lower VD in the SCP and the DCP in COVID-19 patients in the central area, as well as the inner and outer ring [25].

These results were similar to Leyla Hazar et al’s. estimations, which confirmed significantly lower VD in the SCP and DCP one month after patients were discharged following recovery [27].

Another parameter analyzed in this study was VD in the CC plexus. It was significantly lower in the foveal area in COVID-19 patients than in controls, but not in the parafoveal area. Most studies did not estimate VD in the CC plexus. In the study performed by Gonzales-Zamora et al., no alterations in VD in the CC plexus were observed [24].

As all the above-mentioned studies vary significantly in the time of examination (11 days to even 12 weeks after initial symptoms or discharge), it can probably explain the differences in results obtained. There is no consensus yet of when a reliable measurement should be taken, or which mechanisms COVID-19 can affect the vessel density.

In our earlier study, we emphasized the role of the choroid in the nourishment of the outer layers of the retina, part of the optic nerve, and retinal pigment epithelium. The choroid is the only metabolic source in the avascular zone of the macula. Retinal oxygenation is also provided by the choroid, which is one of the most vascularized tissues in the human body. It plays a role in the pathophysiology of many eye and systemic diseases such as anemia or carotid artery stenosis [37].

We suspect that reduced VD in the choriocapillaris in COVID-19 patients can affect the retina. Long-term follow-up is needed to estimate the functional and anatomical consequences on the general and local condition.

### 4.5. Limitations of the Study

Our study has several limitations. It could not be performed during the symptomatic acute phase of COVID-19 due to the emergency condition and risk of contagion. Nineteen COVID-19 patients suffered from hypertension and three from dyslipidemia, so we cannot exclude that part of our findings were related to the general condition. We have no baseline results of the OCT before disease, so we cannot compare these findings. A strong point of the study is a large group consisting of selected patients without the influence of vascular diseases such as diabetes mellitus, examined at the same time point after discharge from the hospital.

## 5. Conclusions

Here we report significant changes in the retinal microvasculature of COVID-19 patients, especially in the most metabolically active regions of the retina. These findings confirm the microvascular involvement of SARS-CoV-2 infection and its possible vascular sequelae. We cannot yet state whether these changes are permanent or transient, but even a minor decrease in the perfusion in the macular area can result in slow degradation of its function. Long-term follow-up studies are required to further evaluate these findings. OCT can be a useful tool to assess the severity of the disease and to determine the effectiveness of the COVID-19 treatment. 

## Figures and Tables

**Table 1:** Demographic, ocular, and systemic characteristics of the study population.

Variables	COVID-19 Patients		Control Group		*p*
	M(SEM)	Me(IQR)	M(SEM)	Me(IQR)	
Men (n; %)	43(68.52)		28(62.22)		0.515
Women	20(31.75)		17(37.78)		
Age	51.33(1.45)	51.00(18.00)	47.76(1.38)	47.00(10.00)	0.087 ^A^
Body height (cm)	173.11(1.20)	176.00(15.00)	171.18(1.07)	170.00(12.00)	0.214 ^B^
Body mass (kg)	85.41(1.97)	88.00(20.00)	78.44(2.33)	78.00(23.00)	0.024 ^A^
BMI (kg/m^2^)	28.41(0.51)	28.00(6.00)	26.77(0.64)	26.50(6.00)	0.047 ^A^
LogMar visual acuity	0.0	0.00	0.0	0.0	-
LogMar reading vision	0.3	0.03	0.3	0.0	-
Spherical equivalent (D)	0.13(0.13)	0.0(2.25)	-0.67(0.13)	0.63(1.37)	<0.001
Axial length	23.55(0.08)	23.45(1.05)	23.35(0.10)	23.45(1.22)	0.111 ^A^
Hypertension	19(30.16%)				
Dyslipidemia	3(4.76%)				
Oxygen therapy	22(34.92%)				

^A^—t Student test; ^B^ Mann–Whitney test. Abbreviations: M—mean; Me—median; SEM—standard error of the mean; IQR—quartiles; D—diopters.

**Table 2:** Comparison of the foveal avascular zone in the superficial capillary plexus (FAZs) and the foveal avascular zone in the deep capillary plexus (FAZd). Bold values denote statistical significance at the *p* < 0.05 level.

FAZ	COVID-19 Patients				Control Group				*p*
	M	SEM	Me	IQR	M	SEM	Me	IQR	
**FAZs (µm^2^)**	326.81	9.77	323.89	126.48	251.03	12.10	243.07	154.03	**0.000**
**FAZd (µm^2^)**	357.76	13.17	344.76	169.33	237.12	11.88	226.41	154.63	**0.000**

**Table 3:** Comparison of the foveal area in the superficial (SCP), deep capillary plexus (DCP), and choriocapillaris (CC) plexus. Bold values denote statistical significance at the *p* < 0.05 level.

F(Foveal)	COVID-19 Patients				Control Group				*p*
	M	SEM	Me	IQR	M	SEM	Me	IQR	
**Superficial Capillary Plexus (%)**	20.69	0.39	20.94	5.16	20.70	0.45	21.14	5.34	0.706
**Deep Capillary Plexus (%)**	17.17	0.43	16.94	6.39	16.93	0.49	16.55	6.92	0.849
**Choriocapillaris (%)**	51.42	0.38	52.05	5.29	52.43	0.47	52.96	6.38	**0.046**

## Data Availability

All data are available from the first author—M.K.

## Supplementary Materials

The following supporting information can be downloaded at: https://www.mdpi.com/article/10.3390/jcm11133621/s1.
