# Supplementary material for: Retinal Microvascular Changes in COVID-19 Bilateral Pneumonia Based on Optical Coherence Tomography Angiography
Source: J Clin Med. 2022 Jun 23;11(13):3621. doi: 10.3390/jcm11133621 (PMC9267319; doi:10.3390/jcm11133621)
Supplement: Supplementary file 1 [file jcm-11-03621-s001.zip › Supplementary Table S1.pdf]

Supplementary Table S1. Comparison of foveal (F) and inner ring superior (IS(IR)), inner nasal (IN(IR)), inner ring inferior (II(IR)), inner ring temporal (IT(IR)), outer ring superior (OS(OR)), outer ring nasal (ON(OR)), outer ring inferior (OI(OR), outer ring temporal (OT(OR)) parameters of RNFL RETINA parameters in COVID-19 patients and age, sex and laterality-matched controls. Mean ±SEM (standard error of the mean) structural OCT values. Bold values denote statistical significance at the $p<0.05$ level.

RNFL RETINA	COVID–19 patients				Control group				p
	M	SEM	Me	IQR	M	SEM	Me	IQR	
F	3.57	0.23	3.00	3.00	3.31	0.24	3.00	2,00	0.333 [A]
IS(IR)	29.18	0.24	29.00	4.00	28.35	0.22	28.00	3,00	**0.024** [B]
IN(IR)	24.28	0.21	25.00	3.00	23.69	0.31	23.00	3,00	**0.018** [B]
II(IR)	29.53	0.32	30.00	3.00	29.16	0.28	29.00	4,00	0.080 [B]
IT(IR)	20.05	0.24	20.00	4.00	19.66	0.30	20.00	4,00	0.326 [B]
OS(OR)	41.83	0.53	41.00	8.50	40.25	0.50	40.00	7,00	0.082 [B]
ON(OR)	52.54	0.76	52.00	10.00	50.36	0.68	50.00	8,00	0.022 [B]
OI(OR)	43.70	0.62	44.00	9.00	42.18	0.60	42.00	7,00	**0.093** [A]
OT(OR)	23.12	0.36	23.00	4.50	22.42	0.28	23.00	4,00	0.217 [B]

[A] – t Student test; [B] Mann – Whitney test.
