# Supplementary material for: Retinal Microvascular Changes in COVID-19 Bilateral Pneumonia Based on Optical Coherence Tomography Angiography
Source: J Clin Med. 2022 Jun 23;11(13):3621. doi: 10.3390/jcm11133621 (PMC9267319; doi:10.3390/jcm11133621)
Supplement: Supplementary file 1 [file jcm-11-03621-s001.zip › Supplementary Table S10.pdf]

Supplementary Table S10. Comparison between men and women in COVID-19 group in OCT parameters: vessel density in foveal (F), inferior (I),superior (S), nasal(N), temporal(T) area in SCP (superficial), DCP (deep capillary plexus), and CC (choriocapillaris) plexus, foveal avascular zone in superficial capillary plexus (FAZs), foveal avascular zone in deep capillary plexus (FAZd), Retinal Nerve Fiber Layer (RNFL) optic disc – inferior area (I), superior area (S),nasal area (N), temporal area (T), Retinal Thickness in inner inferior ring (II), inner superior ring (IS), inner nasal ring (IN), inner temporal ring (IT), outer inferior ring (OI), outer superior ring (OS), outer nasal ring (ON), outer temporal ring (OT), Retinal Nerve Fiber Layer (RNFL) in inner inferior ring (II), inner superior ring (IS), inner nasal ring (IN), inner temporal ring (IT), outer inferior ring (OI), outer superior ring (OS), outer nasal ring (ON), outer temporal ring (OT), Ganglion Cell Layer (GCL) in inner inferior ring (II), inner superior ring (IS), inner nasal ring (IN), inner temporal ring (IT), outer inferior ring (OI), outer superior ring (OS), outer nasal ring (ON), outer temporal ring (OT), Choroidal Thickness (BMCSI) in inner inferior ring (II), inner superior ring (IS), inner nasal ring (IN), inner temporal ring (IT), outer inferior ring (OI), outer superior ring (OS), outer nasal ring (ON), outer temporal ring (OT). Bold values denote statistical significance at the p<0,05 level.

Variables	Men		Women		P
	M(SEM)	Me (IQR)	M(SEM)	Me (IQR)	
OCT A_F_SCP (%)	21.72(0.40)	21.65(4.69)	18.48(0.77)	18.38(5.02)	<0.001 B
OCT A_F_DCP (%)	17.30(0.51)	16.90(4.29)	17.04(0.82)	17.63(5.21)	0.884 B
OCT A_F_CC (%)	52.03(0.44)	52.82(4.29)	50.21(0.71)	49.39(4.61)	0.009 B
OCT A_S_SCP (%)	48.10(0.29)	48.482.92)	48.65(0.46)	48.39(2.27)	0.226 B
OCT A_S_DCP (%)	52.43(0.33)	52.46(4.31)	50.99(0.60)	51.17(5.35)	0.025 A
OCT A_S_CC (%)	53.97(0.21)	54.10(2.43)	54.26(0.38)	54.18(2.84)	0.984 B
OCT A N_SCP (%)	45.03(0.28)	45.17(3.26)	45.28(0.38)	45.46(3.59)	0.526 B
OCTA N_DCP (%)	49.26(0.30)	49.16(3.29)	48.51(0.57)	47.69(4.43)	0.043 B
OCTA N_CC(%)	53.60 (0.23)	53.45(2.48)	53.61(0.33)	53.60(1.82)	0.987 A
OCT A I_SCP (%)	46.98(0.42)	47.53(3.94)	47.70(0.90)	48.41(2.54)	0.039 B
OCT A I_DCP (%)	51.52(0.45)	51.41(5.30)	50.85(0.61)	50.02(4.71)	0.239 B
OCT A I_CC (%)	54.37(0.25)	54.36(2.95)	54.29(0.36)	54.31(3.49)	0.862 A
OCT A T_SCP (%)	46.40(0.26)	46.30(2.84)	47.22(0.31)	47.08(2.72)	0.062 A
OCT A T_DCP (%)	47.93(0.27)	47.88(3.38)	46.87(0.44)	47.26(3.81)	0.036 A
OCT A T_CC (%)	53.87(0.21)	53.91(2.27)	54.22(0.29)	53.91(2.57)	

FAZs(µm2)	303.02(10.28)	299.79(95.58)	377.81(19.65)	372.16(115.08)	<0.00[A]
FAZd(µm2)	342.73(15.68)	329.80(172.35)	380.19(21.99)	363.92(139.94)	0.111 [B]
RNFL OPTIC DISC_S	132.89(1.52)	131.00(19.00)	132.19(2.66)	136.00(14.00)	0.571 [B]
RNFL OPTIC DISC_N	83.34(1.58)	85.00(19.50)	83.46(14.00)	87.00(2.40)	0.966[A]
RNFL OPTIC DISC_I	134.30(2.10)	135.00(16.00)	137.57(15.00)	137.00(2.34)	0.395[B]
RNFL OPTIC DISC_T	79.03(6.22)	71.00(18.50)	72.97(1.48)	73.00(9.00)	0.790[B]
F_RETINAL THICKNESS	249.45(2.93)	254.00(24.00)	236.05(3.25)	239.00(25.00)	<0.001[B]
IS RETINAL THICKNESS	321.23(1.78)	321.50(20.00)	312.32(2.54)	311.00(19.00)	0.001[B]
IN RETINAL THICKNESS	320.87(1.87)	319.00(21.00)	311.08(2.94)	309.00(27.00)	0.005[B]
II RETINAL THICKNESS	318.10(1.67)	317.00(15.00)	309.19(2.72)	307.00(22.00)	0.002[B]
IT RETINAL THICKNESS	308.50(2.30)	309.00(20.00)	295.76(2.86)	295.00(26.00)	<0.001[B]
OS RETINAL THICKNESS	277.94(1.41)	277.50(17.00)	277.38(3.30)	274.00(14.00)	0.096[B]
ON RETINAL THICKNESS	292.28(1.82)	290.50(21.00)	287.22(2.62)	286.00(13.00)	0.082[B]
OI RETINAL THICKNESS	267.65(1.44)	266.00(19.00)	262.78(2.19)	260.00(11.00)	0.023[B]
OT RETINAL THICKNESS	262.22(1.88)	263.50(16.00)	254.43(2.08)	253.00(11.00)	<0.001[B]
RNFL RETINA F	3.88(0.29)	3.00(3.00)	2.92(0.35)	2.00(4.000	0.023[B]
RNFL RETINA_IS	29.39(0.29)	29.00(4.00)	28.62(0.44)	28.00(3.00)	0.142[A]
RNFL RETINA_IN	24.54(0.26)	25.00(3.00)	23.65(0.36)	24.00(3.00)	0.022[B]
RNFL RETINA_II	29.94(0.42)	30.00(3.00)	28.59(0.41)	29.00(4.00)	0.007[B]
RNFL RETINA_IT	20.71(0.26)	21.00(3.00)	18.54(0.43)	18.00(3.000	<0.001[B]
RNFL RETINA_OS	41.22(0.61)	41.00(9.00)	43.16(1.04)	42.00(6.00)	0.171 [B]
RNFL RETINA_ON	52.59(0.98)	53.00(11.00)	52.38(1.19)	52.00(9.00)	0.901[A]
RNFL RETINA_OI	43.65(0.71)	45.00(9.00)	43.81(1.28)	42.00(7.000	0.540[B]
RNFL RETINA_OT	23.60(0.46)	24.00(3.00)	22.03(0.51)	21.00(5.00)	0.022[B]
GCL F	52.62(1.32)	51.50(12.00)	46.16(1.59)	45.00(10.00)	0.002[B]
GCL_IS	123.37(1.04)	124.50(12.00)	121.11(1.51)	123.00(12.000	0.176[B]
GCL_IN	119.11(1.03)	119.50(13.00)	115.84(1.68)	117.00(14.00)	0.103[B]
GCL_II	123.16(1.25)	125.00(10.00)	118.24(3.22)	121.00(18.00)	0.164[B]
GCL_IT	112.34(1.03)	114.00(8.00)	107.08(1.39)	108.00(13.00)	0.002[B]
GCL_OS	105.98(1.01)	106.00(14.00)	107.41(1.58)	107.00(6.00)	0.673[B]

GCL_ON	122.37(1.29)	122.00(12.00)	122.22(1.88)	123.00(11.00)	0.966[B]
GCL_OI	105.57(1.09)	106.50(12.00)	105.78(1.67)	104.00(8.00)	0.692[B]
GCL_OT	94.220.86)	95.00(9.00)	90.54(1.33)	90.00(7.00)	0.005[B]
BMCSI F	271.22(7.76)	265.00(89.00)	257.97(11.97)	242.00(104.00)	0.349[A]
BMCSI IR_IS	272.23(7.05)	275.00(76.00)	260.78(10.66)	247.00(85.00)	0.369[A]
BMCSI IR_IN	253.34(7.32)	246.00(99.00)	243.84(11.97)	245.00(104.00)	0.484[A]
BMCSI IR_II	262.76(7.68)	268.50(88.00)	245.49(11.66)	239.00(112.00)	0.215[A]
BMCSI IR_IT	259.11(7.64)	258.50(87.00)	257.30(11.02)	248.00(83.00)	0.894[A]
BMCSI OR_OS	271.50(6.11)	268.00(79.00)	254.03(10.97)	236.00(76.00)	0.138[F]
BMCSI OR_ON	199.85(6.75)	191.50(88.00)	203.81(13.04)	193.00(121.00)	0.767[A]
BMCSI OR_OI	241.02(7.30)	239.00(79.00)	239.51(12.97)	226.00(104.00)	0.914[A]
BMCSI OR_OT	236.04(6.97)	233.50(75.00)	242.11(9.85)	230.00(81.00)	0.669[B]

[A] – t Student test; [B] – Mann-Whitney test;
