# Supplementary material for: Retinal Microvascular Changes in COVID-19 Bilateral Pneumonia Based on Optical Coherence Tomography Angiography
Source: J Clin Med. 2022 Jun 23;11(13):3621. doi: 10.3390/jcm11133621 (PMC9267319; doi:10.3390/jcm11133621)
Supplement: Supplementary file 1 [file jcm-11-03621-s001.zip › Supplementary Table S2.pdf]

Supplementary Table S2. Comparison of foveal (F) parameters of RNFL RETINA, GCL, BMCSI, RETINAL THICKNESS angiography (OCTA) parameters in COVID-19 patients and age, sex, and laterality-matched controls. Mean ±SEM (standard error of the mean) structural OCT values. Bold values denote statistical significance at the p<0.05 level.

Foveal area	COVID – 19 patients				Control group				p
	M	SEM	Me	IQR	M	SEM	Me	IQR	
RNFL RETINA	3.57	0.23	3.00	3.00	3.31	0.24	3.00	2.00	0.333 [A]
GCL	50.63	1.06	49.50	12.00	49.33	1.11	48.00	16.00	0.485 [A]
BMCSI	266.41	6.49	260.50	95.50	278.58	8.27	273.00	98.00	0.243 [A]
RETINAL THICKNESS	245.39	2.30	249.00	80.00	244.02	2.12	243.00	22.00	0.278 [B]

[A] – t Student test; [B] Mann – Whitney test.
