# Supplementary material for: Retinal Microvascular Changes in COVID-19 Bilateral Pneumonia Based on Optical Coherence Tomography Angiography
Source: J Clin Med. 2022 Jun 23;11(13):3621. doi: 10.3390/jcm11133621 (PMC9267319; doi:10.3390/jcm11133621)
Supplement: Supplementary file 1 [file jcm-11-03621-s001.zip › Supplementary Table S3.pdf]

Supplementary Table S3. Comparison of optic nerve average total RNFL thickness, Average superior and Average inferior RNFL thickness in COVID-19 patients, and age, sex, and laterality-matched controls. Mean ±SEM (standard error of the mean) structural OCT values. Bold values denote statistical significance at the p<0.05 level.

Optic nerve head	COVID– 19 patients				Control group				p
	M	SEM	Me	IQR	M	SEM	Me	IQR	
Average total RNFL thickness	106.30	0.89	106.50	11.00	103.63	1.17	104.00	10.00	0.068 [A]
Average superior RNFL thickness	132.49	1.33	131.50	18.00	127.63	1.43	129.00	17.00	**0.021** [B]
Average inferior RNFL thickness	136.42	1.33	136.00	16.00	131.68	2.01	131.00	20.00	**0.010** [B]
