# Supplementary material for: Retinal Microvascular Changes in COVID-19 Bilateral Pneumonia Based on Optical Coherence Tomography Angiography
Source: J Clin Med. 2022 Jun 23;11(13):3621. doi: 10.3390/jcm11133621 (PMC9267319; doi:10.3390/jcm11133621)
Supplement: Supplementary file 1 [file jcm-11-03621-s001.zip › Supplementary Table S4.pdf]

Supplementary Table S4. Comparison of foveal (F) and inner ring superior (IS(IR)), inner nasal (IN(IR)), inner ring inferior (II(IR)), inner ring temporal (IT(IR)), outer ring superior (OS(OR)), outer ring nasal (ON(OR)), outer ring inferior (OI(OR), outer ring temporal (OT(OR)) parameters of GCL parameters in COVID-19 patients and age, sex, and laterality-matched controls. Mean ±SEM (standard error of the mean) structural OCT values. Bold values denote statistical significance at the p<0.05 level.

GCL	COVID – 19 patients				Control group				p
	M	SEM	Me	IQR	M	SEM	Me	IQR	
F	50.63	1.06	49.50	12.00	49.33	1.11	48.00	16.00	0.485[A]
IS_IR	122.79	0.86	123.50	12.00	119.66	1.09	119.00	12.00	**0.012**[B]
IN_IR	118.21	0.89	119.00	13.00	115.41	1.14	115.00	14.00	**0.023**[B]
II_IR	121.77	1.33	124.00	11.50	120.51	1.17	120.00	12.00	0.081[B]
IT_IR	110.85	0.86	112.00	9.50	108.72	1.00	107.00	12.00	**0.023**[B]
OS_OR	106.47	0.85	106.00	11.00	113.31	9.58	104.00	13.00	0.126[B]
ON_OR	122.41	1.05	122.00	12.00	119.58	1.27	118.00	12.00	0.088[B]
OI_OR	105.72	0.90	106.00	11.50	102.65	1.06	103.00	12.00	**0.029**[A]
OT_OR	93.14	0.73	93.00	9.50	91.47	0.83	91.00	8.00	0.136[A]

[A] – t Student test; [B] Mann – Whitney test.
