# Supplementary material for: Retinal Microvascular Changes in COVID-19 Bilateral Pneumonia Based on Optical Coherence Tomography Angiography
Source: J Clin Med. 2022 Jun 23;11(13):3621. doi: 10.3390/jcm11133621 (PMC9267319; doi:10.3390/jcm11133621)
Supplement: Supplementary file 1 [file jcm-11-03621-s001.zip › Supplementary Table S5.pdf]

Supplementary Table S5. Comparison of OCTA angiography (OCTA) parameters in COVID-19 patients and age, sex, and laterality-matched controls. Mean ±SEM (standard error of the mean) structural OCTA values. Parafoveal area in SCP (superficial), DCP (deep capillary plexus), and CC (choriocapillaris) plexus. Bold values denote statistical significance at the p<0.05 level.

**Parafoveal area**	**COVID – 19 patients**				**Control group**				**p**
	M	SEM	Me	IQR	M	SEM	Me	IQR	
**Superficial Capillary Plexus (%)**	51.95	0.22	52.39	2.81	51.84	0.28	51.85	3.44	0.620^{B}
**Deep Capillary Plexus (%)**	54.29	0.24	54.03	3.81	53.86	0.27	53.95	3.93	0.241^{A}
**Choriocapillaris (%)**	66.86	0.12	66.91	1.50	66.91	0.20	66.97	1.97	0.456^{B}
