# Supplementary material for: Retinal Microvascular Changes in COVID-19 Bilateral Pneumonia Based on Optical Coherence Tomography Angiography
Source: J Clin Med. 2022 Jun 23;11(13):3621. doi: 10.3390/jcm11133621 (PMC9267319; doi:10.3390/jcm11133621)
Supplement: Supplementary file 1 [file jcm-11-03621-s001.zip › Supplementary Table S6.pdf]

Supplementary Table S6. Comparison of OCTA angiography (OCTA) parameters in COVID-19 patients and age, sex, and laterality-matched controls. Mean±SEM (standard error of the mean) structural OCTA values. The superior area in SCP (superficial), DCP (deep capillary plexus), and CC (choriocapillaris) plexus. Bold values denote statistical significance at the p<0,05 level.

**Superior area**	**COVID – 19 patients**				**Control group**				**p**
	M	SEM	Me	IQR	M	SEM	Me	IQR	
**Superficial Capillary Plexus (%)**	48.28	0.25	48.45	2.74	48.23	0.37	48.19	3.84	0.374[B]
**Deep Capillary Plexus (%)**	51.99	0.30	52.10	4.52	52.18	0.40	51.89	4.63	0.688[A]
**Choriocapillaris (%)**	54.06	0.18	54.15	2.42	54.24	0.28	54.23	2.44	0.422[B]
