# Supplementary material for: Retinal Microvascular Changes in COVID-19 Bilateral Pneumonia Based on Optical Coherence Tomography Angiography
Source: J Clin Med. 2022 Jun 23;11(13):3621. doi: 10.3390/jcm11133621 (PMC9267319; doi:10.3390/jcm11133621)
Supplement: Supplementary file 1 [file jcm-11-03621-s001.zip › Supplementary Table S8.pdf]

Supplementary Table S8. Comparison of OCTA angiography (OCTA) parameters in COVID-19 patients and age, sex, and laterality-matched controls. Mean ±SEM (standard error of the mean) structural OCTA values. The temporal area in SCP (superficial), DCP (deep capillary plexus), and CC (choriocapillaris) plexus. Bold values denote statistical significance at the p<0,05 level.

**Temporal area**	**COVID – 19 patients**				**Control group**				**p**
	M	SEM	Me	IQR	M	SEM	Me	IQR	
**Superficial Capillary Plexus (%)**	46.66	0.20	46.60	2.76	46.44	0.24	46.52	3.29	0.538[B]
**Deep Capillary Plexus (%)**	47.61	0.23	47.77	3.33	47.20	0.33	46.93	4.39	0.212[B]
**Choriocapillaris (%)**	53.99	0.17	53.93	2.59	53.50	0.50	54.06	2.43	0.967[B]
