# Supplementary material for: Retinal Microvascular Changes in COVID-19 Bilateral Pneumonia Based on Optical Coherence Tomography Angiography
Source: J Clin Med. 2022 Jun 23;11(13):3621. doi: 10.3390/jcm11133621 (PMC9267319; doi:10.3390/jcm11133621)
Supplement: Supplementary file 1 [file jcm-11-03621-s001.zip › Supplementary Table S9.pdf]

Supplementary Table S9. Comparison of OCTA angiography (OCTA) parameters in COVID-19 patients and age, sex and laterality-matched controls. Mean ±SEM (standard error of the mean) structural OCTA values. The nasal area in SCP (superficial), DCP (deep capillary plexus) and CC (choriocapillaris) plexus. Bold values denote statistical significance at the p<0,05 level.

**Nasal area**	**COVID – 19 patients**				**Control group**				**p**
	M	SEM	Me	IQR	M	SEM	Me	IQR	
**Superficial Capillary Plexus (%)**	45.05	0.23	45.23	3.59	44.99	0.27	45.05	3.57	0.569[B]
**Deep Capillary Plexus (%)**	49.04	0.27	48.60	3.85	48.45	0.32	47.91	4.18	0.160[A]
**Choriocapillaris (%)**	53.61	0.19	53.57	2.26	53.22	0.25	53.32	2.89	0.351[B]
